# Supplementary material for: WordCommentsAnalyzer: A windows software tool for qualitative research
Source: F1000Res. 2018 Sep 4;7:536. Originally published 2018 May 3. [Version 2] doi: 10.12688/f1000research.14819.2 (PMC6137411; doi:10.12688/f1000research.14819.2)
Supplement: Supplementary file 3 [file f1000research-7-17614-s0002.tgz › b2a79496-4ada-47c4-9682-a99ce867836d.zip!/Eight Iranian Health Journals/Yafteh _31.docx]

© Yafteh  . This work is licensed under CC BY 4.0 (https://creativecommons.org/licenses/by/4.0/).

Volume & Issue: دوره 13 - شماره 1

Title: اثرات کوآنزیم Q10 بر میزان لیپیدهای سرم در موش‌های صحرایی نر دیابتی

http://yafte.lums.ac.ir/article-1-387-fa.html

شیوع دیابت در جهان درحال افزایش است. در حال حاضر بیش از 180میلیون نفر مبتلا به دیابت در جهان وجود دارد و‌ تا سال 2030 این تعداد دوبرابر خواهد شد.در دیابت مارکرهای استرس اکسیداتیوافزایش می‌یابد. کوآنزیم Q10 به عنوان یک آنتی‌اکسیدان قوی باعث مهار پراکسیداسیون لیپیدها وحذف رادیکال‌های آزاد می‌شود. دراین مطالعه اثرات کوآنزیم Q10 بر میزان لیپیدهای سرم در موش‌های صحرایی نر دیابتی بررسی شده است. بحث و نتیجه‌گیری: باتوجه به اینکه کوآنزیم Q10 باعث کاهش فاکتورهای لیپیدی سرم می‌شود می‌توان با بررسی بیشتر از آن به عنوان مکمل درمانی دیابت استفاده کرد. یافته‌ها: نتایج بدست آمده نشان داد که کوآنزیم Q10 تری‌گلیسرید، کلسترول،LDL و VLDL نسبت به گروه دیابتی درمان نشده(05/0>p) کاهش می‌دهد ولی افزایش HDL سرم معنی‌دار نبود. مواد و روش‌ها: بیست و یک عدد موش صحرایی نژاد ویستار(200-250 گرم) بطور تصادفی به سه گروه تقسیم شدند(در هرگروه هفت عدد)، گروه اول کنترل، گروه دوم دیابتی و گروه سوم دیابتی درمان شده با کوآنزیم Q10 با دوز ( mg/kg15)؛ گروهای دوم وسوم از طریق تزریق تتراهیدرات آلوکسان ( mg/kg120) دیابتی شدند. بعداز شش هفته درمان از آنها خون تهیه شدو میزان لیپیدهای سرم اندازه گیری شد. داده های بدست آمده با نرم افزار SPSS وآزمون Mann Whitney ارزیابی شد.

Title: بررسی کارائی فرایند ترکیبی انعقاد و اکسیداسیون پیشرفته شبه فنتون در حذفCOD فاضلاب سنتتیک آنتی‌بیوتیک کلاریترومایسین از فاضلاب

http://yafte.lums.ac.ir/article-1-390-fa.html

آنتی بیوتیک‌ها جزو آلاینده‌های مهم محیط‌های آبی به حساب می‌آیند.در این مطالعه حذفCOD آنتی بیوتیک کلاریترومایسن از فاضلاب سنتتیک ازطریق فرایند ترکیبی انعقاد و اکسیداسیون پیشرفته با شبه فنتون مورد بررسی قرار گرفته است مواد و روش‌ها: این تحقیق در مقیاس آزمایشگاهی و به صورت ناپیوسته انجام گرفت. فاضلاب سنتتیک از آنتی بیوتیک کلاریترو مایسین با غلظت 200 میلی‌گرم بر لیتر ساخته شد. شاخصCOD به عنوان پارامتر مورد بررسی در این تحقیق انتخاب گردید. ابتدا فرایند انعقاد بر روی فاضلاب سنتتیک انجام شد و پس از حصول شرایط بهینه برای منعقدکننده مناسب ،پساب این مرحله وارد فرایند اکسیداسیون شبه فنتون گردید. در فرایندشبه فنتون تاثیر تغییرات pH، پراکسید هیدروژن و Fe0 برکارایی حذف COD فاضلاب حاوی آنتی بیوتیک کلاریترو مایسین، مورد ارزیابی قرار گرفت و شرایط بهینه برای هر کدام از پارامتر‌ها تعیین شد. یافته‌ها: براساس نتایج حاصل از این تحقیق، منعقده‌کننده پلی‌آلومنیوم کلراید به عنوان بهترین منعقدکننده انتخاب گردید. برای این منعقدکننده در pH بهینه برابر 7 و بامقدار ماده منعقدکننده برابر 100 میلی‌گرم بر لیتر میزان حذف COD فاضلاب برابر 37/84% حاصل شد. پارامتر‌های اپتیمم در فرایندشبه فنتون، برای حذف کلاریترو مایسین نیز، به ترتیب pH برابر7 ، Fe0برابر 3/0میلی‌مول بر لیتر، پر اکسید هیدروژن 3/0 میلی مول بر لیتر و زمان ماند5/0 ساعت تعیین شد. در ضمن نسبت بهینه H2O2/Fe0 برابر 1 بدست آمد. با اعمال این شرایط میزان حذف CODفاضلاب با فرایند تلفیقی انعقاد و اکسیداسیون پیشرفته با شبه فنتون ، 95/97% تعیین شد. بحث و نتیجه‌گیری: در مجموع با توجه به آزمایشات انجام شده، نتایج آزمایشات حاکی از آن است که فرایند ترکیبی انعقاد و شبه فنتون کارایی مناسبی در حذف COD فاضلاب حاوی آنتی‌بیوتیک دارد. ‏‏‏‏‏‏ ولی کاربرد این روش در صنعت باید مورد بررسی قرار گیرد.

Title: بررسی وضعیت واحد پذیرش بستری در بیمارستان‌های آموزشی دانشگاه علوم پزشکی لرستان در سال 1387

http://yafte.lums.ac.ir/article-1-391-fa.html

مقدمه: واحد پذیرش در هر سازمان بهداشتی و درمانی ورودی و خروجی سیستم را کنترل می‌کند؛ و به کارگیری برخی از فنون مدیریتی مانند تحلیل فرایند، نقش مهمی در شناخت مشکلات این واحد دارد. بنابراین، مطالعه حاضر با هدف بررسی وضعیت واحد پذیرش بستری در بیمارستان‌های آموزشی شهر خرم آباد در سال 1387 انجام شد. مواد و روش‌ها: این پژوهش، مطالعه‌ای توصیفی است که به صورت مقطعی انجام شده است. داده‌ها با استفاده از چک لیست محقق ساخته مبتنی بر استانداردهای واحد پذیرش به روش مشاهده و مصاحبه با صاحبان فرایند گردآوری گردید. اعتبار محتوای آن بر اساس نظر 7 نفر از اساتید و صاحبنظران در این زمینه تایید و برای پایایی از روش آزمون مجدد استفاده گردید. تحلیل فرایند واحد پذیرش بستری نیز در هر یک از بیمارستانها صورت گرفت. داده ها با استفاده از امتیاز بندی، آستانه‌ی تحلیل و شاخص‌های آمار توصیفی و به کمک نرم‌افزار SPSS مورد تحلیل قرار گرفت. اطلاعات جمع آوری شده به صورت جداول آماری تهیه گردید. بحث و نتیجه‌گیری: در مجموع درجه مطلوبیت واحد پذیرش در کلیه بیمارستان‌های مورد مطالعه "متوسط" بود. تهیه و تدوین دستورالعمل‌های خاص واحد پذیرش بستری، به کارگیری نیروی انسانی متخصص و علاقمند، برگزاری دوره های بازآموزی، تخصیص بهینه منابع، تجهیزات و فضای مورد نیاز ،ارزیابی مداوم عملکرد این بخش و انجام اقدامات بهبود فرایند در جهت ارتقا عملکرد این بخش حائز اهمیت است. یافته‌ها: فرایند واحد پذیرش بستری در بیمارستان‌های تحت مطالعه مشابه است و در کلیه بیمارستان‌های مذکور، از بین واحدهای دخیل در این فرایند تنها واحد صندوق از تمرکز مکانی برخوردار می‌باشد. متوسط زمان انتظار بیماران برای پذیرش بستری (50/20+10/19) دقیقه بود. درجه مطلوبیت تسهیلات فیزیکی و فضا ،پرسنل ،روندکاری و خط مشی‌ها ،تجهیزات و سیستم مکانیزه واحد پذیرش بستری در بیمارستان‌های مورد پژوهش با درصد فراوانی (5/35%)در گروه "متوسط" قرار گرفت. بالاترین درصد تطابق با استاندارد مربوط به تجهیزات با 3/43% و کمترین آن مربوط به تسهیلات فیزیکی و فضا با 30% بود.

Title: بررسی مقایسه‌ای تاثیر کرم واژینال گیاه مورت و کلوتریمازول در درمان واژینیت کاندیدایی

http://yafte.lums.ac.ir/article-1-392-fa.html

واژینیت کاندیدایی یکی از رایج‌ترین مشکلات زنان است و سالانه بیش از 10 میلیون زن بدین علت مورد مشاوره و درمان قرار می‌گیرند .به طور شایع داروهای موضعی از جمله گروه آزول در درمان این بیماری به کار گرفته می‌شود،که در مواردی استفاده از این داروها موجب بروز عوارض جانبی و عود مکرر عفونت واژینال می‌گردد.اخیراًً گزارش‌های متعددی در مورد اثرات ضدقارچی و ضدمیکروبی گیاه مورت ارائه شده است.این پژوهش باهدف تعیین اثرکرم واژینال مورت و مقایسه آن با کرم واژینال کلوتریمازول در بیماران مبتلا به واژینیت کاندیدایی انجام شد . مواد و روش‌ها: در این کارآزمایی بالینی دوسوکور تصادفی 80 زن غیرباردار در دو گروه دریافت‌کننده کرم واژینال مورت وکرم واژینال کلوتریمازول قرار گرفتند .علائم و نشانه‌های بالینی و آزمایشگاهی در ابتدای مطالعه و پس از درمان توسط پرسشنامه و فرم مشاهده ثبت گردید .درمان در هر دو گروه به مدت 7 روز انجام شد .داده ها توسط آزمون مجذور کای ،تی تست ،من ویتنی، مک نمار و ویل کاکسون تجزیه و تحلیل گردید . بحث و نتیجه‌گیری: این مطالعه نشان داد اثرات درمانی کرم واژینال مورت نه تنها مشابه کرم کلوتریمازول می‌باشد بلکه در بهبود بعضی از علائم واژینیت کاندیدایی اثر بخشی بیشتری دارد . بنابر این استفاده از این فرآورده را می‌توان برای درمان عفونت کاندیدایی واژن پیشنهاد نمود. یافته‌ها: نتایج پژوهش اختلاف آماری معنی‌داری را در بهبود خارش دستگاه تناسلی و ترشح در گروه مورت نسبت به گروه کلوتریمازول نشان داد(002/0=p).نتایج مثبت کشت اول (یک هفته پس از درمان )در دو گروه مورت و کلوتریمازول (5/7درصددر برابر 5/17 درصد )و کشت دوم(یک ماه پس از درمان)(15درصد در برابر 6/25 درصد )مشابه هم بودند واختلاف آماری معنی‌داری مشاهده نشد .

Title: بررسی تاثیر امگا 3 در درمان افسردگی

http://yafte.lums.ac.ir/article-1-393-fa.html

مقدمه: اختلال افسردگی از شایع‌ترین و ناتوان کننده‌ترین سندرم‌های روانپزشکی است. از آنجا که نتایج تحقیقات حاکی از وجود ارتباط معنی‌داری بین کاهش سطح امگا 3 و خلق افسرده است ، بر آن شدیم تا تاثیر امگا3 بر درمان افسردگی بیماران افسرده را مورد بررسی قرار دهیم . مواد و روش‌ها: مطالعه حاضر یک کارآزمائی بالینی است که بر روی80 بیمار افسرده مراجعه کننده به درمانگاه تخصصی اعصاب و روان انجام گرفت. بیماران بطور کاملا تصادفی یک سوکور در دو گروه تحت درمان با امگا 3 و نورتریپتلین قرار گرفتند. کلیه بیماران در هفته‌های4 ، 8 و 12 مورد بررسی قرار گرفتند. اطلاعات پس از جمع‌آوری مورد تجزیه و تحلیل آماری قرار گرفت. بحث و نتیجه‌گیری: با توجه به یافته‌های تحقیق مبنی بر تاثیر امگا 3 و با عنایت به بی‌ضرر بودن استفاده از این داروها پیشنهادمی‌گردد از داروهای فوق در افسردگی‌های غیرسایکوتیک به عنوان خط اول درمان استفاده گردد. یافته‌ها: نتایج تحقیق نشان داد که میانگین نمره افسردگی بک در هر دو گروه تحت مطالعه در هفته‌های 4 ، 8 و 12 به میزان قابل توجهی کاهش داشته و میزان عملکرد بیماران بهبود یافته است. آزمون آماری Repeted measures تفاوت معنی‌داری (001/0 >p ) در میانگین نمرات سطح افسردگی و عملکرد بیماران دو گروه قبل از مداخله درمانی و هفته‌های 4 ،8 و 12 ( ماههای اول ، دوم و سوم ) بعد از شروع درمان، نشان داد. بین تاثیر داروها در دو گروه تفاوت معنی‌داری بدست نیامد.

Title: مقایسه تجویز کلومیفن ودگزامتازون باکلومیفن به تنهایی در القاء تخمک‌گذاری و حاملگی در خانم‌های نابارور با مشکل تخمدان پلی‌کیستیک در شهرستان خرم‌آباد

http://yafte.lums.ac.ir/article-1-395-fa.html

مقدمه: نازایی یکی از مهم‌ترین مشکلات در بین زوج‌ها می‌باشد که معمولا یک نفر از هر 7 نفر زوج را تحت تاثیر قرار می‌دهدکه هدف از این مطالعه ارزیابی اثر دگزامتازون و کلومیفن سیترات در درمان تخمدان پلی‌کیستیک با سطح طبیعی دهیدرواپی آندرستندیون سولفات در القاء تخمک‌گذاری بود.که با توجه به آمارهای متعدد که عدم تخمک‌گذاری در زنان نابارور تا 40% نیز دیده شده بنابراین اهمیت روش‌های تحریک تخمک‌گذاری اهمیت خاصی دارد. مواد و روش‌ها: در این مطالعه که یک کارآزمایی بالینی آینده‌نگر می‌باشد 120 بیمار نابارور با تشخیص سندرم پلی‌کیستیک بطور تصادفی از تاریخ 1/6/85 لغایت 1/10/87 به مدت 28 ماه در دو گروه دریافت‌کننده کلومیفن و دگزامتازون(گروه اول) و دریافت‌کننده کلومیفن و پلاسبو مورد بررسی قرار‌گرفتند. که در این مطالعه با استفاده از پرسشنامه‌ای که روایی و پایایی آن به تایید رسیده بود سن بیماران، مدت نازایی، الگوی ‌قاعدگی، سطح دهیدرواپی آندرستندیون سولفات –هیرسوتیسم –شاخص توده بدنی –تعدادفولیکولهای بالاتر از 18 میلی متر –تخمک‌گذاری و حاملگی مورد بررسی قرارگرفت. یافته‌ها: نتایج مطالعه نشان داد هیچگونه اختلاف معنی‌دار بین دو گروه از نظر سن، مدت نازایی، شاخص توده‌بدنی،الگوی قاعدگی و سطح سرمی دهیدرو‌اپی آندرستندیون سولفات وجود نداشت همچنین بین دو گروه از نظر هیرسوتیسم تفاوت معنی‌داری وجود نداشت .تعداد فولیکولهای بالاتر از 18 میلی‌متر بطور معنی‌دار در گروه دریافت‌کننده دگزامتازون و کلومیفن (059/0=p)بیشتر بود همچنین میزان تخمک‌گذاری و حاملگی نیز بطور معنی‌دار در این گروه نسبت به گروه دریافت‌کننده کلومیفن و پلاسبو بیشتر بود. بحث و نتیجه‌گیری: القاء تخمک‌گذاری با اضافه نمودن دگزامتازون به درمان کلومیفن سیترات روشی بسیار موثر، ارزان و ساده در درمان تخمدان پلی‌کیستیک و القاء تخمک‌گذاری و حاملگی می‌باشد.

Title: بررسی اثر ویتامین‌های C‏وE در پیشگیری از مسمومیت حاملگی در زنان باردار شکم اول

http://yafte.lums.ac.ir/article-1-397-fa.html

مسمومیت حاملگی از اختلالات پرفشاری دوره بارداری است که یکی از علل عمده مرگ و میر و موربیدیتی مادر و جنین است. گزارشات اخیر حاکی از آن است که رادیکال‌های آزاد القاء‌کننده آسیب اندوتلیال سلول ممکن است در ایجاد بیماری نقش داشته باشد. هدف از این مطالعه تعیین اثر آنتی‌اکسیدان‌هایی مانند ویتامین C , E در پیشگیری از مسمومیت حاملگی می باشد. این مقاله استخراج شده از پایان‌نامه دانشجویی می‌باشد. بحث و نتیجه‌گیری: براساس یافته‌های این پژوهش استفاده از آنتی اکسیدان‌هایی مانند ویتامین C و E احتمال بروز پره‌اکلامپسی به طور واضحی کاهش می‌یابد، هم‌چنین می‌توانند باعث کاهش فشار خون شوند. در مجموع مصرف آنها در دوران حاملگی توصیه می‌شود. یافته‌ها: میزان بروز مسمومیت حاملگی در گروه شاهد 14 نفر (5/17 درصد) و در گروه مورد 4 نفر (5 درصد) بود (05/0>p). میانگین فشار سیستول در گروه مورد قبل و بعد از مداخله به ترتیب3/8 37/99 و 25/13 12/106 میلی متر جیوه و در گروه شاهد 8/7 43/99 و 1/19 44/12 میلی‌متر جیوه بود (001/0p<). میانگین فشار دیاستول در گروه مورد قبل و بعد از مداخله 5 7/62 و 8/8 9/64 میلی‌متر جیوه و در گروه شاهد 7/4 7/62 و 2/13 1/68 میلی‌متر جیوه بود (001/0p<). مواد و روش‌ها: در این مطالعه کارآزمایی بالینی، 160 خانم باردار 38-18 ساله شکم اول که هیچ‌کدام از عوامل خطر مسمومیت حاملگی را نداشتند به دو گروه تقسیم شدند. در گروه اول ویتامین E روزانه IU 400 و ویتامین C روزانه 1000 میلی‌گرم همراه قرص آهن در هفته 24-20 حاملگی تا پایان بارداری تجویز شد و گروه دوم این ویتامین‌ها به رژیم غذایی آنها اضافه نشده و فقط قرص آهن مصرف کردند. در پایان اطلاعات جمع‌آوری شده با استفاده از روش‌های آماری توصیفی( میانگین، انحراف معیار و توزیع فراوانی ) و آمار استنباطی ( T-Test) و با استفاده از نرم افزار(15SPSS(ver مورد تجزیه و تحلیل قرار گرفت.

Title: اتیولوژی و پاتوژنز آنتی‌اسپرم آنتی‌بادی

http://yafte.lums.ac.ir/article-1-398-fa.html

آنتی‌اسپرم آنتی‌بادی‌ها (ASA) در مردان و زنان ایجاد می‌شوند و ممکن است به طور قابل توجهی باروری را معیوب نمایند. در این مورد، بیضه یک مکان امن ایمونولوژیک است که در آنجا آنتی‌ژن‌های سلول ژرم از حمله خودایمنی محافظت می‌شوند. با این وجود، در نتیجه شکسته شدن سد خونی- بیضه‌ای حاصل از آسیب بیضه‌ای یا در نتیجه ضربه به اپیدیدیم یا وازدفران اغلب پروتئین‌های بیضه‌ای در طول برخوردهای ایمونولوژیکی منجر به تشکیل ASA در سرم خون، پلاسمای سمن یا سطح غشا اسپرم، اتوآنتی ژنیک می‌شوند. همچنین گزارش شده است که ASA با التهاب، کریپتورکیدیسم، واریکوسل و مداخله جراحی در اندام‌های تناسلی ارتباط دارد. ASA ممکن است در عملکردهای مختلف اسپرم که برای روندهای باروری ضروری هستند ایجاد اختلال نماید. این مقاله مروری به افزایش فهم ما از مکانیسم‌های اختصاصی که پاسخ خودایمنی به اسپرم را برمی‌انگیزند و پاتوژنز ASA که به ناباروری با واسطه آنتی‌بادی منجر می‌گردد کمک می‌کند.

Title: تأثیرحرکات اصلاحی منظم بر ناهنجاری‌های عضلانی- اسکلتی دختران دانش‌آموز شهر خرم‌آباد

http://yafte.lums.ac.ir/article-1-401-fa.html

شناخت بدشکلی‌ها وحرکات اصلاحی شاخه‌ای از تربیت بدنی به شمار می‌رود که درصدد اصلاح و برطرف کردن ضعف‌ها و ناهنجاری‌های مختلف عضلانی، ارگانیکی و هماهنگی و تعادل با استفاده از حرکات و برنامه‌های دقیق و اصولی ورزش می باشد. هدف از تحقیق حاضر بررسی تأثیر حرکات اصلاحی منظم بر ناهنجاری‌های اسکلتی(اسکولیوز، پای‌پرانتزی، افتادگی شانه ها و کف پای صاف ) دختران دانش‌آموز شهر خرم‌آباد در سال 89-88 بود. مطالعه حاضر از نوع نیمه‌تجربی است، تعداد 160 نفر از دختران دانش‌آموز شهرستان خرم‌آباد به عنوان نمونه آماری انتخاب شدند. ابزار مورد استفاده شامل صفحه شطرنجی، پودی‌اسکوپ، تست‌های مرتبط با سنجش ناهنجاری‌ها، شاقول و متر نواری بود.پرسشنامه مورد استفاده به صورت پیش‌آزمون و پس‌آزمون بررسی تمرینات کششی و قدرتی بمدت 24 جلسه در طول 2 ماه انجام و تجزیه و تحلیل داده‌ها با استفاده از آزمون کوواریانس انجام شد. تمرینات اصلاحی اعمال شده در این تحقیق می‌تواند راه‌حلی مناسب، ارزان و بدون عارضه‌ی جانبی جهت برطرف نمودن ناهنجاری‌های اسکلتی عضلانی روشی مفید، ارزشمند و ارزان در درمان افرادی باشد که در سنین پایین به ناهنجاری‌های عملکردی ستون فقرات مبتلا می-شوند و می‌تواند از بروز مشکلات و ناهنجاری‌های دوران پیری بکاهد میزان ناهنجاری افتادگی شانه، کف پای صاف و پای‌پرانتزی در دختران 12 ساله پس از انجام تمرینات دارای کاهش معنی‌داری بود( به ترتیب 001/0 p< و 96/7 = F ، 001/0 p< و 1/100 = F و 001/0 p< و 63/27 = F ) اما در مورد اسکولیوز تفاوت معنی‌داری مشاهده نشد(001/0 p< و32/3 = F) . در مورد افتادگی شانه، کف پای‌صاف و پای‌پرانتزی در دختران 13 ساله پس از انجام تمرینات دارای کاهش معنی‌داری بود( به ترتیب 001./0 p< و96/7= F و 001/0 p< و 1/100 = F و 001/0 p< و 63/27 = F) اما در مورد اسکولیوز تفاوت معنی-داری مشاهده نشد(001/0 p< و32/3 = F) .میزان ناهنجاری افتادگی شانه، کف پای‌صاف و پای‌پرانتزی در دختران 14 ساله پس از انجام تمرینات دارای کاهش معنی‌داری بود( به ترتیب 001/0 p< و 96/7 = F و 001/0 p< و 1/100 = F و 001/0 P< و 63/27 = F) اما در مورد اسکولیوز تفاوت معنی‌داری مشاهده نشد(001/0 p< و32/3 = F).

Title: بررسی اثر ایزوترتینوئین خوراکی بر سطح فولات سرم

http://yafte.lums.ac.ir/article-1-405-fa.html

آکنه و ولگاریس یکی از شایعترین بیماری‌های پوستی محسوب می‌شود که طی سال‌های اخیر برای درمان انواع شدید و مقاوم به درمان آن از ایزوترتینوئین خوراکی استفاده‌ی زیادی می‌شود. با توجه به اختلاف نظر در مورد اثر ترتینوئین خوراکی بر روی کاهش اسید‌فولیک سرم و نیز با توجه به اینکه، کاهش اسید‌فولیک می‌تواند منجر به بیماری‌های مهم دیگری شود ، این تحقیق به منظور بررسی اثر ایزوترتینوئین خوراکی بر روی سطحی سرمی اسید‌فولیک طی سال‌های 87و88 در شهرستان خرم‌آباد انجام شد. نتایج این تحقیق نشان می‌دهد که مصرف 30 روزه ایزوترتینوئین خوراکی باعث کاهش سطح اسید‌فولیک سرم می‌شود که با توجه به نقش مهم اسیدفولیک لازم است پژوهش دیگری با روزهای بالاتر و مدت طولانی‌تر و همچنین بر روی سایر فاکتور‌های موثر بر متابولیسم اسیدفولیک انجام شود. همچنین مصرف مکمل اسیدفولیک حین مصرف ایزوترتینوئین پیشنهاد می‌شود. میزان اسیدفولیک سرم قبل از شروع داروnmol/lit 42/9± 75/26 و بعد از 30 روز به nmol/lit 42/8 ± 64/23 این کاهش با (008/0=p )معنی‌دار بود . مطالعه بصورت مداخله‌ای بر روی 61 بیمار مبتلا به آکنه وولگاریس شدید و متوسط مقاوم به درمان شامل 38 زن و 23 مرد با سن 6±6/23 سال انجام شد. طی 30 روز به میزان mg/ kg5/0 روزانه ایزوترتینوئین خوراکی برای افراد تجویز شد و از آنها خواسته شد که داروهای موثر بر سطح اسیدفولیک سرم استفاده نکنند و رژیم غذایی خود را ثابت نگه دارند.سطح اسید فولیک سرم قبل از شروع دارو و پس از یک ماه سنجیده شد و نتایج با آزمون Tزوج مورد مقایسه قرار گرفت.

Title: بررسی میزان سدیم،پتاسیم و اسمولاریته پلاسما در بیماران ضربه مغزی در بخش مراقبت‌های ویژه اعصاب

http://yafte.lums.ac.ir/article-1-406-fa.html

از اهداف مایع درمانی بیماران با ادم مغزی حفظ جریان خون مغز و جلوگیری از تغییرات زیاد در اسمولاریته پلاسما و هیپرگلیسمی می‌باشد. تحقیقات نشان داده است مانیتول باعث افزایش اسمولاریته پلاسما بصورت وابسته به دوز و در نتیجه باعث کاهش محتوای آب مغز شده است. همچنین ضمن مطالعاتی در مورد تاثیر سالین هیپرتونیک،کاهشICP را بدلیل اثر اسموتیک آن دانسته‌اند. مطالعه حاضر جهت تعیین سطح سرمی سدیم، پتاسیم و اسمولاریته سرم در شدت‌های مختلف ضایعات مغزی انجام شد. بین تغییرات اسمولاریته و میانگین الکترولیت‌ها با GCS رابطه‌ای وجود نداشت که احتمال تاثیر عوامل دیگر به غیر از اسمولاریته بر روی شدت ضایعات مغزی، نظیر وضعیت ABG ، نیاز به تهویه مکانیکی بعضی از بیماران، پوزیشن، عوامل دارویی و مداخلات درمانی دیگر، درجه حرارت بیمار و ماندن نمونه‌های خونی در آزمایشگاه را خاطر‌نشان می‌سازد. تعداد بیماران به ترتیب روز‌های متوالی از نظر شدت ضایعات مغزی از شدید به خفیف روند رو به کاهش را نشان می‌داد. میانگین اسمولاریته گروه دارای ضایعه عصبی متوسط، از نظر آماری اختلاف معنی‌داری را نسبت به گروه خفیف و شدید نشان داد (3./. = P ). میانگین غلظت سدیم و پتاسیم از نظر آماری اختلاف معنی‌داری در هر3 گروه GCS را نشان نداد این مطالعه تجربی ـ مشاهده‌ای آینده نگر بر روی تعداد 57 مورد بیمار دچار ضربه مغزی بستری درICU اعصاب انجام گردید.سطح هوشیاری بیماران به همراه آزمایشات سدیم و پتاسیم در ساعت 6 صبح روزانه تا مدت 4 روز مشخص شد. اسمولاریته بیماران طبق فرمول Osm=2Na+BUN/2.8+G/18 محاسبه گردید. بیماران بر اساسGCS و روز بستری به 3و4 گروه تقسیم شدند. در هر 24 ساعت 15-13 = GCS در گروه خفیف، 12-9 = GCS در گروه متوسط و8 ≥GCS در گروه شدید از نظر شدت ضایعات مغزی قرار می‌گرفت. یافته‌های پژوهش با استفاده از نرم افزار Spss و آزمون‌های آماری مورد تجزیه و تحلیل قرار گرفتند

Title: کنترل کیفی آزمایش انگل‌شناسی مدفوع در آزمایشگاه‌های تشخیص طبی شهر تبریز

http://yafte.lums.ac.ir/article-1-407-fa.html

هدف از برنامه کنترل کیفیت، ایجاد اعتماد در کارکنان آزمایشگاه و پزشکان به نتایج آزمایشگاهی و در نتیجه افزایش اطمینان به دستاوردهای آزمایشگاهی می‌باشد. کنترل و تضمین کیفیت در واقع به معنی بالا بردن سطح کیفیت بوده و در برگیرنده تمام آزمون‌هایی است که منجر به بالا بردن سطح کاری و بازده آزمایشگاه می‌شود و حداقل هزینه برای جامعه و حداقل زمان برای کارکنان آزمایشگاه را در پی داشته باشد. هدف از این مطالعه ارزیابی و تعیین صحت و دقت نتایج آزمایشگاه‌های تشخیص طبی شهر تبریز بوده است. ‌به منظور ارتقاء کیفیت در آزمایشگاه‌های تشخیص طبی، پایش مستمر و کنترل کیفی آنها با روش‌های استاندارد امری ضروری است. با حدود اطمینان 95 درصد، حساسیت کلی نتایج آزمایش مدفوع برای تشخیص تخم کرمها 62 درصد، برای کیست تک یاخته‌ها 22 درصد و برای تشخیص تروفوزوئیت تک یاخته‌ها 8/0 درصد بود. در این مطالعه که یک مطالعه سنجش مهارت از نوع گذشته نگر می‌باشد، 790 نمونه مدفوع به طور تصادفی از نمونه‌های پذیرش شده در آزمایشگاه‌های تشخیص طبی شهر تبریز با روش‌های استاندارد مورد بررسی مجدد قرارگرفت. نتایج با نرم‌افزار SPSS و آزمون Student t-test ونیز فرمول‌های حساسیت و دقت، مورد تجزیه و تحلیل قرار گرفت.

Title: تاثیر حاد ضربه‌های وارد بر سر ناشی از یک مسابقه بوکس آماتور بر نوار مغزی (EEG)

http://yafte.lums.ac.ir/article-1-409-fa.html

ضربه‌های وارد بر سر می‌توانند موجب اثرات حادی بر سیستم عصبی شوند. ازاین‌رو، در مجامع پزشکی‌ورزشی، گروهی بوکس را همراه با عوارض جسمانی و عصب‌شناختی می‌دانند. اما، عده‌ای نیز معتقدند که پیامدهای جانبی این ورزش بیش از سایر ورزش‌ها نیست. بر این اساس، پژوهش حاضر با هدف بررسی تأثیر حاد ضربه‌های وارد برسر ناشی از یک مسابقه بوکس آماتور بر نوار مغزی، انجام شد. میان نوار مغزی بوکسورها و کشتی‌گیران تفاوت معنی‌داری مشاهده گردید(000/0=p). به طوری‌که پس از انجام یک نوبت مسابقه بوکس، 8 مورد نوار مغزی غیرطبیعی در بوکسورها مشاهده شد، حال آن‌که تمامی نوارهای مغزی گروه کنترل (کشتی‌گیران) طبیعی بود. آزمودنی‌ها در دو گروه 20 نفره قرار گرفتند: گروه اول(گروه تجربی): از 110 بوکسور آماتور و گروه دوم( گروه کنترل): از 230 کشتی‌گیر شهرستان همدان. برای درک اختلال مغزی، از روش مصاحبه بوسیله پزشک استفاده گردید. سپس قبل و 30 تا 45 دقیقه پس از انجام مسابقه از آزمودنی‌ها نوار مغزی بعمل آمد. بنظر می‌رسد که ماهیت ضربه به سر در ورزش بوکس، موجب اختلال در جریانات الکتریکی بافت مغز می‌شود. این تنش که با آسیب‌های حاد مغزی ملایم همراه است و در ترسیم الکتروآنسفالوگرافی بوکسورها به چشم می‌خورد، خطرناک بوده و می‌تواند زمینه بروز عارضه‌های بعدی را فراهم کند.

Title: مقایسه یافته‌های سونوگرافی کیسه صفرا در کودکان و نوجوانان با و بدون اضافه وزن

http://yafte.lums.ac.ir/article-1-410-fa.html

با توجه به ارتباط اضافه‌وزن و چاقی با اختلالات سیستم صفراوی در بزرگسالان، دراین مطالعه یافته‌های سونوگرافیک سیستم صفراوی در کودکان و نوجوانان با و بدون اضافه وزن مورد مقایسه قرارگرفت. در این مطالعه مقطعی 372 کودک و نوجوان 18-6 ساله شهراصفهان براساس شاخص توده بدنی در دو گروه نرمال و دارای اضافه وزن قرارگرفتند. وجوداختلالات سیستم صفراوی توسط سونوگرافی در دو گروه مقایسه شد. میانگین سنی افراد مورد مطالعه 2/3±6/12 سال بود. میانگین قطر مجاری صفراوی در گروه دارای شاخص توده بدنی طبیعی 1/1±7/2 ودر گروه شاخص توده بدنی بالا 3/1±3 میلی‌متر بود. بین میانگین قطر مجاری صفراوی و شاخص توده بدنی رابطه معنی‌داری وجودداشت(04/0p=). بین سن نمونه‌هاو قطر مجاری صفراوی همبستگی مستقیم به میزان 13/0 وجود داشت که معنی‌دار بود(01/0p=).وضعیت کیسه صفرا در همگی طبیعی بود. بین شاخص توده بدنی و ضخامت جدار کیسه صفرا نیز رابطه آماری معنی‌داری وجود نداشت. با تشخیص زودرس بیماری‌های صفراوی مرتبط با اضافه وزن و چاقی در کودکان و نوجوانان می‌توان مانع پیشرفت بیماری شد و عوارض را کاهش داد.

Title: بررسی رابطه بین رضایت زناشویی با مشکلات جسمی دوران بارداری و عوامل مرتبط با آن

http://yafte.lums.ac.ir/article-1-411-fa.html

اختلال‌های روان فیزیولوژیایی نشانه‌های جسمی حقیقی هستند که در نتیجه عوامل هیجانی ایجاد شده یا ممکن است در اثر آن‌ها وخیم‌تر شوند. اگر واکنش به استرس ما را مستعد بیماری جسمانی می‌سازد پس رویدادهای استرس زای مکرر باید با بیماری در ارتباط باشد. بارداری و زایمان برای اکثر زنان با استرس زیادی همراه است میزان رضایتمندی زناشویی در این دوران می تواند بر شدت استرس مادر تاثیرگذار بوده و مشکلات جسمانی و روانی او را تحت تاثیر قرار دهد به همین دلیل پژوهش حاضر به بررسی رابطه بین رضایت زناشویی و مشکلات دوران بارداری می پردازد. همچنین ارتباط بین عوامل روان شناختی با شدت مشکلات جسمی دوران بارداری می پردازد. برای بررسی این پژوهش، روش توصیفی از نوع همبستگی استفاده شده است. جامعه آماری این پژوهش شامل کلیه زنان باردار شهر خرم آباد می باشد که در تابستان 1388 به مراکز بهداشتی- درمانی مراجعه کرده اند. برای جمع آوری اطلاعات، از بین 15 مرکز بهداشتی- درمانی شهر خرم آباد 2 مرکز به صورت تصادفی انتخاب شدند و سپس از هر مرکز با کمک ماما 20 نفر (در کل 40 نفر) با استفاده از روش نمونه گیری تصادفی ساده انتخاب شدند و همزمان پرسش نامه رضایت زناشویی اینریچ با 11 خرده مقیاس، و پرسشنامه مشکلات جسمی شایع دوران بارداری بر روی آزمودنی ها اجرا شد. اطلاعات به دست آمده با استفاده از روش همبستگی، رگرسیون، آزمون t و ANOVA مورد تجزیه و تحلیل قرار گرفت. 1-بین مشکلات شایع دوران بارداری و نمره کل رضایت زناشویی رابطه معکوس و معناداری وجود دارد. 2-بین مشکلات شایع دوران دوران بارداری و متغببر هایی از قبیل سن زن و تفاوت سنی بین زن و مرد رابطه مثبت و معناداری وجود دارد. 3-زنان دارای مقاطع تحصیلی فوق لیسانس و سیکل درمقایسه با زنان دارای مدارک تحصیلی دیپلم و لیسانس از رضایت زناشویی پایین تری برخوردارند.بر اساس نتایج به دست آمده و با توجه به اهمیت تاثیر استرس دوران بارداری بر سلامت جسمانی و روانی مادر، به زوجین توصیه می شود نهایت تلاش خود را در جهت افزایش رضایتمندی زناشویی به منظور کاهش استرس مادران و به دنبال آن مشکلات جسمی دوران بارداری به کار ببرند.
